# Das Recht auf Teilhabe thematisieren: Potenziale der Sozialpädagogischen Nutzer_innenforschung am Beispiel der Analyse von Räumen der Besonderung in der Heimerziehung

**DOI:** 10.1007/s12054-023-00577-5

**Published:** 2023-04-11

**Authors:** Kathrin Aghamiri, Anne van Rießen

**Affiliations:** 1 FH Münster, Münster, Deutschland; 2 Düsseldorf, Deutschland

**Keywords:** Räume der Besonderung, Sozialpädagogische Nutzer_innenforschung, Partizipation, Beschädigungen, Spaces of particularity, Socio-pedagogical user research, Participation, Damages

## Abstract

Rechte von Kindern und Jugendlichen im Kontext der stationären öffentlichen Jugendhilfe werden immer wieder missachtet. So werden im Spannungsfeld von Hilfe und Kontrolle Wohngruppen aus der Perspektive der dort lebenden jungen Menschen zu „Räumen der Besonderung“. Die Forschungsperspektive der Sozialpädagogischen Nutzer_innenforschung bietet eine Möglichkeit, das Recht der Kinder und Jugendlichen auf einen eigensinnigen Standpunkt zu realisieren.

## Abstract (en)

The rights of children and young people in the context of inpatient public youth welfare are repeatedly disregarded. Thus, in the area of tension between help and control, residential groups become “spaces of specialization” from the perspective of the young people living there. The research perspective of social pedagogical user research offers a possibility to realize the right of children and adolescents to an obstinate point of view.

Nutzer_innen Sozialer Arbeit, darunter auch Kinder und Jugendliche, haben Rechte, die im Arbeitsalltag oft missachtet werden. Sie müssen darin bestärkt werden, ihre Rechte und Ansprüche sichtbar zu machen. Es geht vor allem darum, Beschädigungen durch Soziale Arbeit zu vermeiden und Räume der Besonderung abzubauen.

Alltage in professionellen pädagogischen Wohneinrichtungen wie Heimen oder Wohngruppen finden generell in einer institutionell gerahmten Sphäre, in einem „Spannungsfeld von Öffentlichkeit und Privatheit“ (Meuth 2021, S. 443), statt. Damit können spezifische *Besonderungen und Schädigungen* für die dort lebenden Menschen einhergehen (vgl. u. a. van Rießen und Aghamiri 2023; Gundrum und Oelerich 2021). Zur Konkretisierung aus Nutzer_innensicht zu Beginn das Zitat eines jungen Menschen, der in der Kinder- und Jugendhilfe als ‚Systemsprenger‘ etikettiert wurde:

„B1: […] WIR SIND NICHT diese Systemsprenger, WIR SIND GEZEICHNET worden, […] WIR HABEN gewisse ERLEBNISSE erlebt, […] wo sich niemand REINversetzen kann und UNS als SYSTEMsprenger dastehen zu lassen, ist eine tut mir Leid, wenn ich das so ausdrücke, eine EHRENLOSE Sache. […] Es hat nichts- nicht damit zu tun, dass wir das System EXTRA SPRENGEN, dass wir extra die Regelungen BRECHEN, sondern es hat DAMIT zu tun, dass […] die uns einfach diese FREIHEIT genommen hat! [I: Ja.] So und diese FREIHEIT möchten wir einfach WIEDER haben! […] man muss erst RECHT so was solche Leute angeht, einen gewissen FREIRAUM LASSEN, ansonsten bricht es immer und immer wieder.“ (Z. 1044–1093)^1^

Der Jugendliche beschreibt hier eindrücklich das Spannungsfeld, das sich in den institutionalisierten Wohnformen für ihn zwischen Regelanforderungen und seinem Bedürfnis nach Selbstbestimmung ergibt. So vergleichen junge Menschen z. B. ihre Situation in Wohngruppen und Heimen mit bisherigen, alltäglichen Erfahrungen des Wohnens mit Familie oder Freund_innen und beurteilen das Leben in der Wohngruppe in der Folge als „anders“ (vgl. Jepkens et al. 2020). Im Heim zu leben, heißt letztlich besonderen Regeln unterworfen zu sein, in denen die Ambivalenzen sozialarbeiterischen Handelns *Hilfe versus Kontrolle, Partizipation versus Disziplinierung* oder *Schutzort versus totale Institution* (vgl. Trede und Winkler 2006) nachdrücklich spürbar werden (können). In der Figur der *Besonderung* machen wir diese Antinomien anschaulich (van Rießen und Aghamiri 2023). Es zeigt sich, dass das Recht auf Privatheit, auf soziale Teilhabe und Selbstbestimmung mitunter willkürlich dem pädagogischen Machtverhältnis zum Opfer fällt (Stork und Aghamiri 2016). Fachkräfte erziehen, schützen und „lieben“ dann über die Köpfe der jungen Menschen hinweg. Soziale Arbeit läuft in institutionellen Wohnformen Gefahr, Klientifizierung und Exklusion zu betreiben und durch Besonderungen das Recht junger Menschen auf Teilhabe zu behindern bzw. „the right to express [their own] views“ (UNICEF 2023: Art. 12 UN-KRK) aus dem Blick zu verlieren. Das Anliegen unseres Beitrags ist es aufzuzeigen, wie die Perspektive der Sozialpädagogischen Nutzer_innenforschung dazu beitragen kann, den jungen Menschen eine Stimme in den öffentlichen Fachdebatten zu geben. Wir argumentieren dies im Sinne einer „konstitutionellen Pädagogik“ wie sie bereits Janusz Korczak beschrieben hat (1929/1992, S. 353): eine Pädagogik, die jungen Menschen nicht deshalb kein Unrecht tut, weil sie ihnen zugewandt erscheint, sondern weil es verbindliche Rechte und öffentliche Sphären von Mitbestimmung und Möglichkeiten der Einflussnahme gibt (Aghamiri und Hansen 2014). Unsere Annahme ist, dass Sozialpädagogische Nutzer_innenforschung (van Rießen und Jepkens 2020; Oelerich und Schaarschuch 2005) die Standpunkte und Ansprüche der Nutzer_innen Sozialer Arbeit öffentlich und damit verhandelbar machen kann (Aghamiri und Enders 2022).

Dafür werden wir im Folgenden zunächst die Perspektive der Sozialpädagogischen Nutzer_innenforschung skizzieren. Anschließend veranschaulichen wir exemplarisch am Beispiel der stationären Kinder- und Jugendhilfe, wie diese Einrichtungen aus der Perspektive der Kinder und Jugendlichen aufgrund unzureichender Umsetzung von Teilhaberechten im Alltag zu „Räumen der Besonderung“ (van Rießen und Aghamiri 2023) werden. Der Beitrag schließt mit einem Fazit, das die zentrale Bedeutung der Perspektive der Nutzer_innen deutlich macht und das Potenzial einer subjektorientierten Forschung für eine Rechtebasierte Soziale Arbeit begründet.

## Subjektorientierte Perspektive auf Angebote Sozialer Arbeit

Über das Verhältnis von institutionalisierten Wohnformen der Sozialen Arbeit und ihrer professionellen Rahmung, wie Heime oder Wohngruppen, und derjenigen, die diese Einrichtungen in Anspruch nehmen (müssen), kann unterschiedlich nachgedacht werden. Um Zugänge zu einer Rechtebasierten Sozialen Arbeit zu eröffnen, werden wir dies im Folgenden aus der Perspektive der Inanspruchnehmenden, also der jungen Menschen selbst, tun. Dazu greifen wir auf die Sozialpädagogische Nutzer_innenforschung (Oelerich und Schaarschuch 2005) zurück, die sich auf die Beantwortung der Frage konzentriert, welchen *Nutzen* die Angebote Sozialer Arbeit sowohl in ihrer inhaltlichen wie auch in ihrer prozessualen Ausrichtung aus Sicht der Nutzenden haben. Stellt man sich der Frage nach dem Nutzen Sozialer Arbeit aus der Perspektive der Inanspruchnehmenden, gerät damit – und das ist gewollt! – auch der Nicht-Nutzen Sozialer Arbeit in den Fokus der Analyse. Die Frage nach dem Nutzen geht zugleich immer mit der Frage nach den nutzenstrukturierenden Bedingungen einher, unter denen ein potenzieller Nutzen erst realisiert bzw. eben auch *nicht* realisiert werden kann. Denn der potenzielle Nutzen konkretisiert sich stets mittels sozialer Dienstleistungen in spezifischen gesellschaftlichen und institutionellen Kontexten und damit auch in Abhängigkeit zu diesen. In dieser Perspektive geht es somit darum, die nutzenfördernden wie die nutzenlimitierenden Bedingungen auf den differenzierten Ebenen zu analysieren, um davon ableitend deutlich zu machen, welche Barrieren und Begrenzungen eine Nutzung ver- oder behindern. Letztlich werden so auch Beschädigungen und *Besonderungen*, die sich im Kontext der Nutzung ergeben können, sichtbar (vgl. die Beiträge in van Rießen und Jepkens 2020; van Rießen und Aghamiri 2023; Gundrum und Oelerich 2021).

Damit schließt die Sozialpädagogische Nutzer_innenforschung an eine emanzipatorisch ausgerichtete Weiterentwicklung Sozialer Arbeit an, denn die Analysen offenbaren, ob und unter welchen Bedingungen die Angebote Sozialer Arbeit eine Option darstellen, mittels derer es den Inanspruchnehmenden möglich ist, ein selbstbestimmte(re)s bzw. gelingenderes Leben zu führen (vgl. Schaarschuch 2008). Somit ist das Beurteilungskriterium der Angebote Sozialer Arbeit aus dieser Perspektive die (Ge)Brauchbarkeit für das eigene Leben, die konkret nur von den Nutzenden selbst beantwortet werden kann. Diese starke Fokussierung auf die Nutzenden ergibt sich aus einem Verständnis von Sozialer Arbeit als personenbezogene soziale Dienstleistung (Schaarschuch 1999), die ohne die selbsttätige Aneignung und Nutzung der Inanspruchnehmenden nicht denk- und verstehbar ist. Denn diese erscheinen als die eigentlichen Produzent_innen der sozialen Dienstleistungen, da sie ihre Lebenszusammenhänge, Krisen und Probleme selbst bearbeiten (müssen). Angebote Sozialer Arbeit stellen demzufolge eine Ressource dar, welche von ihnen im Hinblick auf ein selbstbestimmte(re)s Leben angeeignet werden können. Grundlage dafür ist, dass die nutzenstrukturierenden Bedingungen, Zugangsmöglichkeiten, Beziehungsangebote, Partizipationsgelegenheiten o. ä., eine solche Nutzung zulassen.

Vor dem Hintergrund dieser Forschungsperspektive geben wir im Weiteren exemplarische Einblicke in das alltägliche Erleben von Jugendlichen, die in professionellen stationären Wohnformen leben (müssen). Dabei greifen wir auf empirische Praxisforschung zurück, die im Rahmen von Lehr-Lern-Forschungsprojekten in den Jahren 2020 bis 2022 entstanden, also einhergehend mit der Ausbreitung des SARS-CoV-2-Virus und den damit verbundenen ordnungsrechtlichen Maßnahmen (vgl. Aghamiri et al. 2022). Hierbei zeigt sich, dass diese Maßnahmen, die zunächst zwar *alle* Menschen in ihren Möglichkeiten der Selbst- und Mitbestimmung einschränkend trafen, zusätzlich sehr besondere bzw. *besondernde* Auswirkungen auf die Wahrnehmung ihrer Teilhaberechte durch die jungen Menschen hatten, die in institutionalisierten Wohngruppen leben.

## Das Recht auf Partizipation in der Kinder- und Jugendhilfe

Heimeinrichtungen für Kinder und Jugendliche gehören zu den ältesten Angeboten der Sozialen Arbeit (Kappeler und Hering 2017). Rechtlich geregelt ist dieser Teil der Kinder- und Jugendhilfe als „Heimerziehung, sonstige betreute Wohnform“ in § 34 SGB VIII. Heimerziehung stellt dabei „einen institutionalisierten sozialpädagogischen Ort [dar], an dem ein organisierter Alltag für jene jungen Menschen gestaltet wird, die diesen Alltag aus sozialen und politischen sowie familiären, persönlichen und individuellen Gründen nicht in ihren bisherigen familialen Beziehungsstrukturen verbringen sollen oder können“ (Pluto et al. 2020, S. 7). Um sicherzustellen, dass erstens Kinder und Jugendliche sowohl ihre Perspektive in allen sie betreffenden Angelegenheiten formulieren können und zweitens Verfahren und Strukturen so entwickelt und verankert werden, dass die Interessen und Anliegen der jungen Menschen unabhängig von der individuellen Haltung der jeweiligen Fachkräfte berücksichtigt werden, sind die Rechte für junge Menschen gesetzlich verankert. So ist in der UN-Kinderrechtskonvention (Artikel 12) das grundsätzliche Recht auf freie Meinungsäußerung und der angemessenen Berücksichtigung der Meinung festgelegt (UNICEF 2023). Die entsprechende gesetzliche Grundlage in Deutschland ist das Sozialgesetzbuch VIII, das grundsätzlich vorschreibt, Kinder und Jugendliche an allen sie betreffenden Entscheidungen in der Kinder- und Jugendhilfe zu beteiligen (§ 8 SGB VIII) – mit dem Blick auf die Hilfen zur Erziehung auch an der Entwicklung der Hilfeplanung (§ 36 SGB VIII) – sowie Möglichkeiten der Beschwerde bereit zu stellen (§ 45(2)). Einrichtungen der Kinder- und Jugendhilfe müssen dazu in ihrer Konzeption darlegen, wie sie die Selbstvertretung und Partizipation von Kindern und Jugendlichen sicherstellen. Ferner wird Beteiligung sowohl im politischen Diskurs als auch in der fachwissenschaftlichen Debatte Sozialer Arbeit als grundlegend angesehen (vgl. u. a. Stork und Aghamiri 2016; Aghamiri und Hansen 2014). Partizipation kann dabei als zentrales Handlungsprinzip Sozialer Arbeit verstanden werden, das die Subjektwerdung in Gemeinschaften zum Ziel hat. Dabei ist Partizipation nicht allein auf Teilhabe oder Teilnahme zu beschränken, sondern bedeutet als demokratisches Kernelement „Entscheidungen, die das eigene Leben und das Leben der Gemeinschaft betreffen, zu teilen und gemeinsam Lösungen für Problem zu finden“ (Schröder 1995, S. 14).

## Schädigung als Folge von Nicht-Beteiligung

Während Partizipation zwar gesetzlich verankert und pädagogisch, politisch wie wissenschaftlich unterhintergehbarer Bezugspunkt ist, zeigen aktuelle empirische Studien, ausgehend von der Sozialpädagogischen Nutzer_innenforschung, dass eine konsequente partizipative Alltagspraxis nicht nur keine Regel darstellt, sondern die Nichtbeachtung partizipativer Ansprüche auch mit weiterführenden *Schädigungen* einhergehen kann (Gundrum und Oelerich 2021, S. 175 ff.). Aus Sicht der ‚zu Beteiligenden‘ ist die (gesetzlich verpflichtende!) Partizipation zentral für die Erfahrung das eigene Leben gelingend gestalten zu können. Als Beispiel mag die Aussage einer Jugendlichen aus der eingangs zitierten studentischen Studie von Hubel (2020) dienen:

„Alex: Also wie gesagt ich- ich glaube WIRKLICH, dass (…) ich SELBER entscheiden kann, was weiß ich, wann ich esse, was ich koche, so ob ich jetzt hier mit anderen Leuten was zu tun haben möchte oder NICHT. So ich kann es alles SELBER entscheiden so […] Ich glaube wirklich, dass es der größte Punkt ist und auch weil früher immer über meinen Kopf halt hinweg entschieden wurde, so dass mir das deswegen ganz GUT tut, so einfach zu wissen ich kann selber entscheiden, was und wann ich was mache, so.“ (Z. 263–274)

Sowohl die hier zu Wort kommende Jugendliche als auch die jungen Leute bei Gundrum und Oelerich (2021) beschreiben ausgehend von ihren Erfahrungen in der Kinder- und Jugendhilfe, in ihrer gesellschaftlichen Teilhabe explizit und nachhaltig eingeschränkt worden zu sein. Eine umfassende Beteiligung, die es den Jugendlichen ermöglichen sollte, am Alltag sowie am Hilfeprozess zu partizipieren und sich einzubringen, führt dagegen zu mehr Selbstwirksamkeit und Anerkennung. Nur mit und durch Beteiligung ist es möglich, dass sich der Hilfeprozess an den subjektiven Erfahrungen, der gegenwärtigen Situation sowie den subjektiven Zielstellungen der Einzelnen ausrichten kann (van Rießen 2016; Stork und Aghamiri 2016). Wird also das Recht auf Partizipation verwehrt, unterläuft und konterkariert die Heimerziehung mitunter ihren eigentlichen Auftrag: die Förderung der Entwicklung und Vorbereitung auf ein selbstständiges Leben (§ 34 SGB VIII).

## Räume der Besonderung

Wenn das Recht auf Selbst- und Mitbestimmung schon in Alltagssituationen der Kinder- und Jugendhilfe immer wieder gefährdet erscheint, spitzen Krisenszenarien das Ausmaß der Nicht-Beteiligung von Kindern und Jugendlichen noch einmal zu. Spezifisch einhergehend mit der Ausbreitung des SARS-CoV 2‑Virus und den damit verbundenen ordnungsrechtlichen Maßnahmen, wird deutlich, dass das Recht auf Beteiligung in vielen stationären Einrichtungen der Kinder- und Jugendhilfe ausgesetzt war (vgl. van Rießen und Aghamiri 2023) Während die meisten Jugendlichen davon berichteten, dass Partizipation während der ordnungspolitischen Maßnahmen vor allen Dingen im Freundeskreis und Zuhause stattfand, zeigen empirische Analysen, ausgehend von der Sozialpädagogischen Nutzer_innenforschung, dass Kinder und Jugendliche in stationären Einrichtungen kaum Spielräume der Beteiligung im Hinblick auf die sie betreffenden Regelungen hatten. Dies erlebten sie als spezifische Differenz zu Freund_innen in Familien (van Rießen und Aghamiri 2023):

„Ja, also bei uns ist es ja jetzt zum Beispiel so, dass wenn wir erkältet sind oder Schnupfen haben, müssen wir 48 h auf unserem Zimmer bleiben und dürfen es nicht verlassen (Anna, 16 Jahre).“ (ebd.)

Das öffentlich verantwortete Wohnen schafft auf diese Weise ‚Räume der Besonderung‘ – auch indem den jungen Menschen häusliche, in der Pandemie ohnehin bereits eingeschränkte Möglichkeiten der Selbst- und Mitbestimmung verweigert wurden. Während die professionellen Fachkräfte hier vor allem den Corona-Schutz in einem beruflichen Setting fokussierten, zeigt die Nutzer_innenforschung, welche Spannungsfelder sich diesbezüglich für die Jugendlichen ergab, die in der Wohngruppe ihr *zu Hause* haben. Institutionalisierte Wohnformen können totalitäre Züge annehmen, wenn Definitions- und Gestaltungsmacht allein bei den Fachkräften verbleiben und die jungen Menschen keine Möglichkeit haben, nach außen zu treten. Gerade unter pandemischen Bedingungen erschien diese Gefahr besonders hoch. Eine empirische Perspektive, die die Nutzer_innen in den Blick nimmt, ermöglicht dagegen ein eigenständiges ‚Qualitätsurteil‘ aus eben der Perspektive der Nutzer_innen im Hinblick auf die Angebote Sozialer Arbeit und der darin eingebetteten Verpflichtung zur Partizipation.

## Die zentrale Bedeutung der Nutzer_innenperspektive

Ob Partizipation als Recht auf Teilhabe und Beschwerde in der Praxis eingelöst wird, kann letztlich nicht aus einer professionell-normativen Perspektive bemessen werden. Das Recht auf Beteiligung in *allen* die jungen Menschen betreffenden Angelegenheiten schließt eine stellvertretende Einschätzung durch die Fachkräfte aus. Die Aneignung von Partizipationsgelegenheiten, die Ansprüche und das Bedürfnis nach Selbstbestimmung oder die auf vielfältige Weise vorgebrachten Interessenbekundungen sind nur zu realisieren, wenn jene, die Angebote Sozialer Arbeit nutzen (müssen), sich selbst zeigen oder äußern können. Sozialpädagogische Nutzer_innenforschung kann dazu beitragen, diese Perspektive systematisch zugänglich zu machen und sie in den fachlichen Diskurs einzubringen. Dabei sollte die Sozialpädagogische Nutzer_innenforschung sich allerdings ebenfalls mit den Bedingungen einer Rechtebasierten Sozialen Arbeit auseinandersetzen. Wie werden Äußerungen von Inanspruchnehmenden ermöglicht? Wer „darf“ oder soll sich eigentlich äußern? Wie können Begriffe und Interessen „übersetzt“ oder kommunikativ validiert werden? Nutzer_innenforschung kann die Partizipationsansprüche der Nutzer_innen nämlich ebenfalls willkürlich auswählen, missbrauchen oder umdeuten. Von daher stellt sich im Zirkelschluss die Frage nach partizipativen Ansätzen und Möglichkeiten einer Rechtebasierten Sozialforschung selbst. Partizipation müsste in diesem Sinne auch für Forschungsbemühungen im Kontext Sozialer Arbeit als Recht auf Mitwirkung konkretisiert werden.

## Footnotes

1 Das Zitat stammt aus den Interviewdaten von Annika Hubel (2023): Systemsprenger*innen? – Wie erleben betroffene junge Menschen die Bedingungen innerhalb der Kinder- und Jugendhilfe? Münster: unveröffentlichte Bachelorarbeit. Die Arbeit wurde im Sinne der Sozialpädagogischen Nutzer_innenforschung erstellt. Vielen Dank für das Zurverfügungstellen des Zitats.
